# A case of chronic ossified hematoma presented as a skull lesion: A literature review on two rare conditions, cephalhematoma, and intradiploic hematoma

**DOI:** 10.1002/ccr3.6934

**Published:** 2023-02-08

**Authors:** Arad Iranmehr, Mohammad Kazem Sarpoolaki, Mehdi Zeinalizadeh

**Affiliations:** ^1^ Department of Neurosurgery, Imam Khomeini Hospital Complex Tehran University of Medical Sciences Tehran Iran; ^2^ Department of Neurosurgery, Sina Hospital Tehran University of Medical Sciences Tehran Iran

**Keywords:** birth injury, cephalhematoma, head injury, head trauma, pediatric neurosurgery

## Abstract

Cephalhematoma is a frequent condition in newborn infants due to birth‐related trauma, but ossified cephalhematoma (OCH) is a rare condition, especially when it presents as a skull lesion in the older pediatric population. Chronic intradiploic hematoma (CIH) is another rare condition caused by an organized hematoma between the inner and outer tables of the skull. Differentiating CIH from OCH could be difficult for young neurosurgeons. We present an 18‐month‐old girl with an OCH presented as a skull lesion, which was managed with craniectomy and en‐bloc excision of the organized hematoma. This manuscript discusses the differences between OCH and CIH in diagnosis and management.

## Graphical abstract

Ossified cephalhematoma (OCH) could represent a skull lesion. Past medical history of patients could be useful to differentiate this condition from neoplastic or congenital lesions of the skull. OCH can be managed with craniectomy and en‐bloc excision of the organized hematoma.

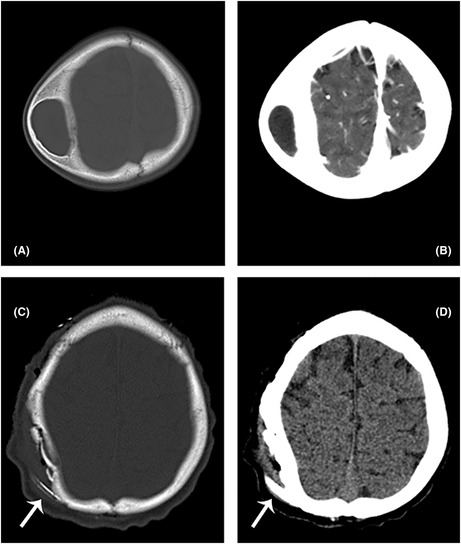

## 1 BACKGROUND

Cephalhematoma is a frequent condition in newborn infants due to birth‐related trauma,^1^ but ossified cephalhematoma (OCH) is a rare condition, especially when it presents as a skull lesion in the older pediatric population.^2^ OCH is located under the periosteum (pericranium), and the pericranium plays a crucial role in enveloping the organized hematoma with ossified tissue.^3^ The presence of a soft fluctuant mass just after birth is the key to the diagnosis of OCH.^4^

Chronic intradiploic hematoma (CIH) is another rare condition caused by an organized hematoma between the inner and outer tables of the skull. Differentiating CIH from OCH could be difficult for young neurosurgeons. CIH presents as a slow‐growing skull mass. The exact pathogenesis is unclear, but repetitive bleeding in the intradiploic space initiated by trauma may be the leading cause.^3^

We present a case of OCH presented as a skull lesion managed with craniectomy and en‐bloc excision of the organized hematoma. We will discuss the differences between OCH and CIH in diagnosis and management.

## 2 CASE REPORT

**FIGURE 1:**
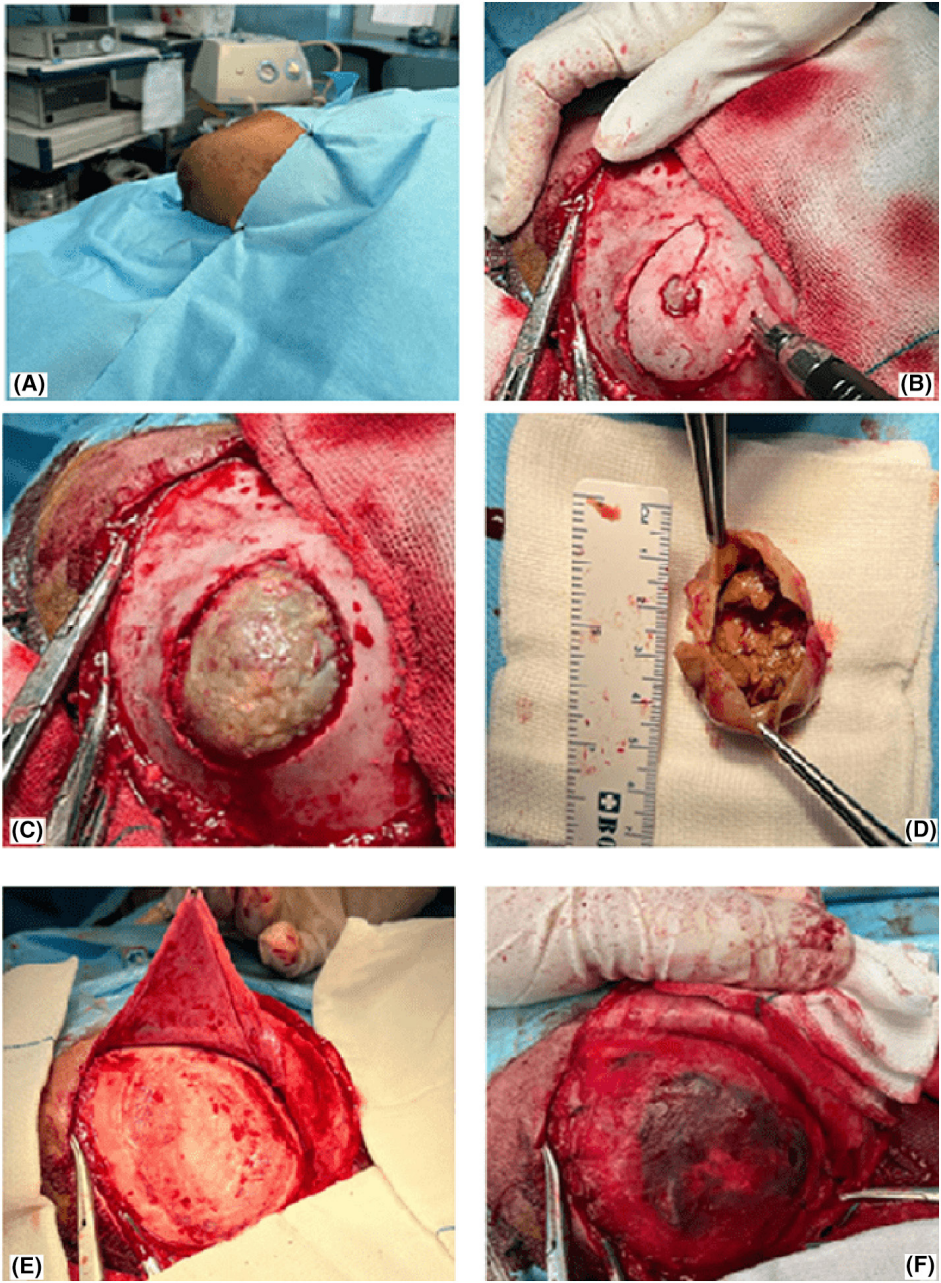
Intraoperative steps of the surgery; (A) the lesion before surgery, (B) the external ossified layer craniectomy, (C) the lesion and its pseudo‐membrane after removing the external ossified layer, (D) size and the contents of the lesion, (E) the inner skull after removing the lesion and smoothening the borders, as well as the harvested pericranium (white star), (F) the final view after reconstruction.

An 18‐month‐old girl presented with a nontender slow‐growing skull lesion in the right parietal region. The lesion was round, and bony textured on touch (Figure 1A). The neurological examination was normal. There was no medical complaint about the lesion other than a cosmetic problem. The patient was born by standard vaginal delivery (NVD) to a nulliparous mother. Based on the mother's report, the baby suffered bilateral parietal soft masses just after birth. Although the left‐sided lesion vanished over time, the bulge on the other side grew for 18 months.

**FIGURE 2:**
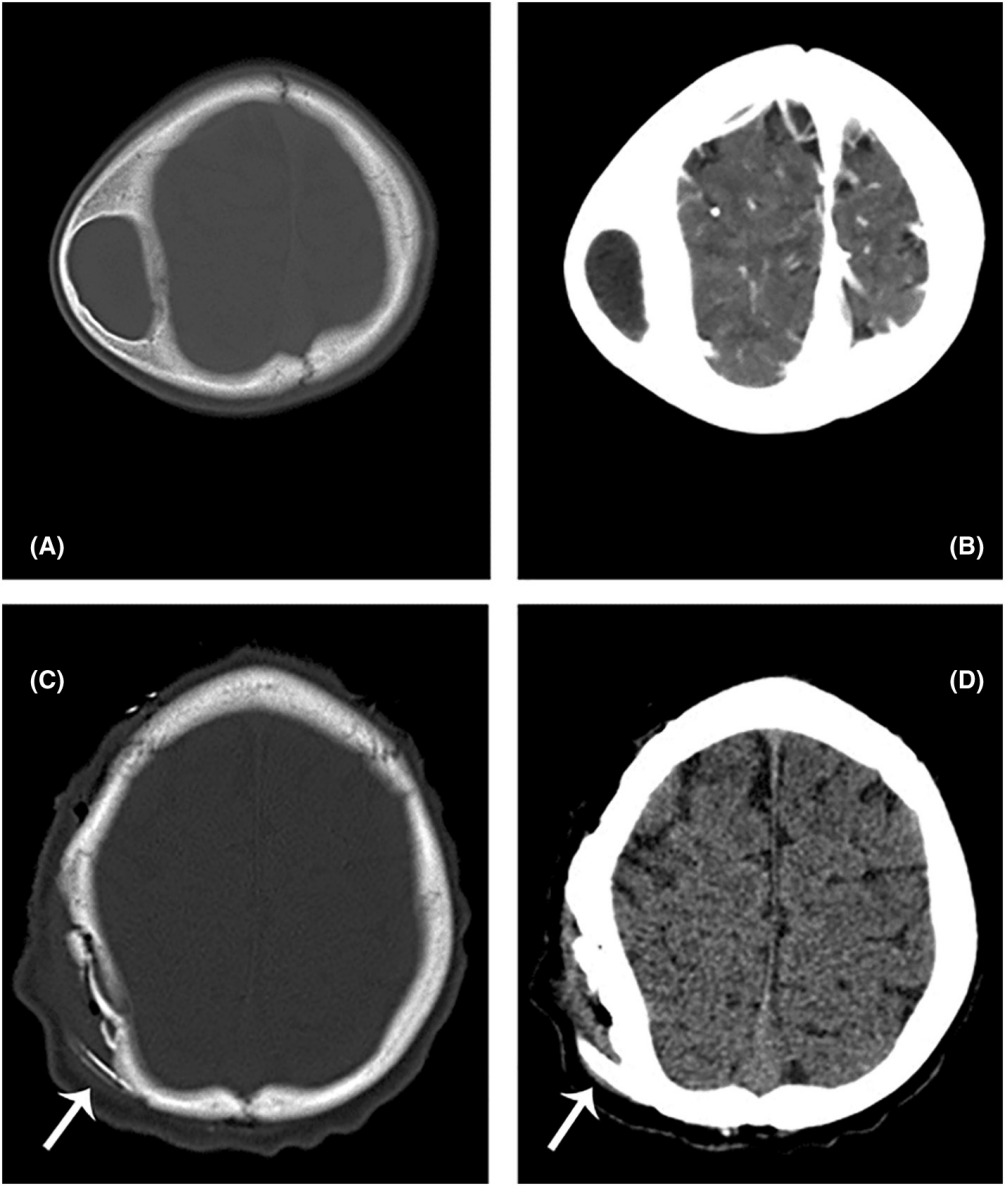
Pre‐ and postoperative images. (A) Axial bone view CT scan of the lesion, (B) axial post‐contrast CT scan of the lesion without any enhancement, (C) Postoperative axial bone and brain (D) view CT scan. Arrows show the drain tube.

On the preoperative examination, it was approximately 7*7*5 cm. The routine laboratory tests were normal without any coagulopathy. Brain computed tomography scan (CT‐scan) with a bone view showed a hypodense expanded mass between the outer and inner tables of the skull in the parietal region (Figure 2A) without any enhancement after contrast administration (Figure 2B). The patient's history and CT scan were characteristic of OCH, so we decided against performing an MRI. Under general anesthesia, the skin was incised semicircularly around the lesion in the supine position by turning the head 60 degrees to the left. Pericranium was dissected from the skin flap to be used for final cranial reconstruction.

**FIGURE 3:**
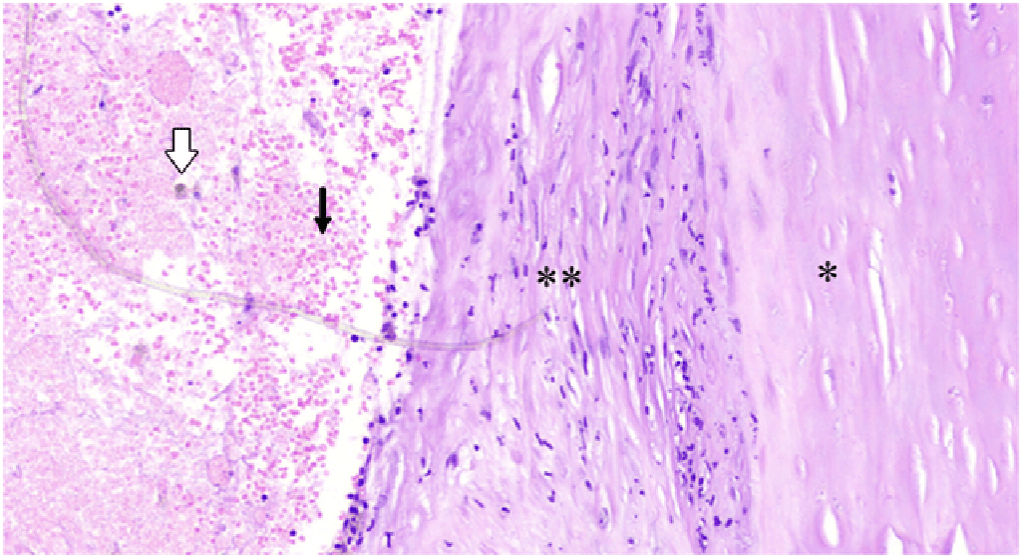
Histopathologic images of the lesion showing RBCs (black arrow), hemosiderin‐laden macrophages (white arrow) indicating old hematoma under the periosteum(asterisk), and fibrotic tissue (double asterisk).

We performed a craniectomy after placing a burr hole over the ossified overlying layer on top of the lesion and blunt dissection over the pseudo‐membrane. (Figure 1B,C). Subsequently, the organized hematoma was dissected circumferentially from the cranial bone (Figure 1D). After en‐block hematoma removal, the uneven lateral borders of the skull were smoothened using a high‐speed drill to maintain the proper shape of the skull (Figure 1E). Finally, we reconstructed the surgical site utilizing a layer of bone chips (harvested from the outer layer of the hematoma) and an overlying pericranium for better future cranial bone shaping and remodeling (Figure 1F). The postoperative course was uneventful. Figure 2C,D shows the normal contour of the skull just postoperation. Histopathologic examination confirmed an organized hematoma with hemosiderin deposition and calcification in line with OCH (Figure 3).

## 3 DISCUSSION

Birth‐related traumas could cause various cranial hematomas in neonates.^5^ Cephalhematoma is among the most common hematomas, but OCH is a rare condition. According to previous reports, approximately 20 percent of cephalhematomas undergo calcification, but most resolve after conservative follow‐up.^2^ When found at older ages, OCH could be mistaken for other ossified skull lesions.^1^ OCH can resemble a depressed fracture that requires different management.^6^ The pericranium covers the hematoma and, in older patients, causes the ossified outlining layer to reach the external table, which could be not distinguished with histological examinations. CIH is another rare pathology that could be mistaken for OCH. The exact etiology is still unknown, but the use of anticoagulants, trauma in birth, and shunt surgeries have all been mentioned in the literature.^7^ Usually, CIH resolves spontaneously, but if it is not, a connective tissue surrounds the hematoma and forms a (pseudo) capsule.^8^ On conventional radiography and CT scan, the typical presentation of a CIH is a well‐circumscribed, expanded intra‐skull lesion with or without surrounding sclerosis with varying attenuations and internal enhancing components after contrast injection.^9^ Table 1 shows the previously reported cases of CIH in patients with confirmed histopathological diagnosis and without coagulopathies. The critical points for differentiating these two conditions could be obtained by a previous history of a soft fluctuant mass just after birth.^4^ Imaging could be helpful in cases where previous diagnostic hints are not available. In OCH cases, the contour of the underlying skull remains normal, but in CIH cases, narrowing of the inner and outer tables of the skull could be seen.^3^ Preoperative imaging is vital to correct diagnosis and treatment; MRI and CT scans are complementary, but in some cases, as in our patient, with a history of birth trauma, the CT scan may be diagnostic, and MRI is not mandatory.

**TABLE 1:** Demonstrating published case reports with CIH. We have excluded the patients with intradiploic hematoma that were finally diagnosed as giant cell reparative granuloma, hemophilic pseudotumor.

Author/Year	Age/Gender	History of trauma	Location	Management
Yausa et al./ 1992^10^	20 years old/ Male	Minor trauma	Parietal	Craniotomy and cranioplasty with methyl meta‐acrylate
Sato et al./ 1994^11^	20 years old/ Male	Minor trauma	Parietal	Craniotomy and cranioplasty with methyl meta‐acrylate
Uemura et al./ 1997^12^	32 years old/ Male	Minor trauma	Frontal	Excision (no data available for reconstruction technique)
Yucesoy et al./ 1999^13^	25 days old/ Male	Birth trauma	Parietal	External table craniectomy (the external table was destroyed)
Mobbs et al./ 2000^14^	3 years old/ Male	Major trauma	Parietal	Curettage following aspiration with extended burr hole
Goel et al./ 2000^15^	58 years old/ Male	Minor trauma	Frontal	Craniectomy without cranioplasty
Batista et al./ 2011^16^	45 years old/ Male	Recurrent trauma	Parietal and occipital	Craniotomy and cranioplasty with non‐autologous material
Tokmak et al./ 2015^7^	16 years old/ Male	Minor trauma	Frontal	Excision (no data available for reconstruction technique)
	64 years old/ Male	Minor trauma	Frontal	Curettage (no data available for reconstruction technique)
Luo et al./ 2017^17^	50 days old/ Male	Birth trauma	Parietal	Craniotomy and cranioplasty with autologous bone
Park et al./ 2020^9^	54 years old/ Male	Major trauma	Parietal	Craniectomy (no data available for reconstruction technique)

The surgical management of these two conditions could be different. In CIH patients, the inner table could need to be repaired with a cranioplasty. Still, in OCH patients, due to the excellent contour of the underlying skull, there is no need for cranioplasty, and an en‐block excision could be obtained with a simple craniectomy as we did in our case.^1^, ^3^ Therefore, we suggest craniectomy with en‐bloc organized hematoma removal as the treatment of choice for OCH. High speed‐drill could be used to smoothen the borders of the underlying bone to give the skull a proper shape, as we did in this case. On the contrary, in CIH patients with the destruction of the inner table, a craniotomy followed by a cranioplasty is a better option.

## 4 CONCLUSION

Ossified cephalhematoma is a rare condition that should be differentiated from other neoplastic or congenital lesions of the skull in the pediatric population. OCH can be managed with craniectomy and en‐bloc excision of the organized hematoma without entering into the epidural space. Previous knowledge about these lesions could be helpful to un‐experienced neurosurgeons for proper diagnosis and management.

## AUTHOR CONTRIBUTIONS

**Arad Iranmehr:** Conceptualization; methodology; visualization; writing – original draft; writing – review and editing. **Mohammad Kazem Sarpoolaki:** Conceptualization; visualization; writing – original draft; writing – review and editing. **Mehdi Zeinalizadeh:** Conceptualization; methodology; supervision; visualization; writing – original draft.

## FUNDING INFORMATION

None.

## CONFLICT OF INTEREST STATEMENT

The authors declare that there are no conflicts of interest in this study, not previously presented.

## CONSENT

Written informed consent was obtained from the patient to publish this report under the journal's patient consent policy.

## Data Availability

All of the data related to this manuscript are available by request from the corresponding author. None of the authors listed in the manuscript are employed by a government agency that has a primary function other than research and/or education.
